# Outro Ator no Aumento da Circulação Colateral no Coração – Outro Potencial Alvo Terapêutico na Medicina Cardiovascular?

**DOI:** 10.36660/abc.20220558

**Published:** 2022-08-25

**Authors:** Luis Henrique Wolff Gowdak

**Affiliations:** 1 Hospital das Clínicas Faculdade de Medicina Universidade de São Paulo São Paulo SP Brasil Instituto do Coração do Hospital das Clínicas da Faculdade de Medicina da Universidade de São Paulo, São Paulo, SP – Brasil

**Keywords:** Circulação Colateral, Neovascularização Fisiológica, Adropina, Isquemia Miocárdica, Doença Arterial Coronariana

Richard Lower, de Amsterdã, chamou a atenção em 1669 pela primeira vez para os canais que conectam as artérias coronárias direita e esquerda. O anatomista suíço Albrecht von Haller demonstrou essas anastomoses dissecando as artérias coronárias. Assim, há séculos, a presença de uma rede colateral bem desenvolvida fornecendo suprimento sanguíneo para um miocárdio subperfundido ganhou interesse significativo na fisiologia e fisiopatologia e, posteriormente, na terapêutica.^1^ Muitas questões mantiveram os cientistas trabalhando incansavelmente para desvendar os principais atores que favorecem o desenvolvimento de vasos colaterais no coração.^2^

A circulação colateral coronariana é uma rede pré-formada de anastomoses imaturas que conectam o território suprido por uma artéria coronária epicárdica com o suprido por outra. Em 1959, Pitt et al.,^1^ estudaram a prevalência de anastomoses coronárias interarteriais em setenta e cinco corações obtidos de autópsia. Dos 15 corações normais, apenas um (6%) apresentou tais anastomoses. Nos casos com doença arterial coronariana oclusiva, fibrose do miocárdio ou infarto, as anastomoses foram encontradas em 75% a 100% dos casos. Ficou claro, desde o início, que a presença de períodos sustentados de isquemia tecidual era um pré-requisito para incitar o estabelecimento de circulação colateral.

Por outro lado, muitos pacientes com angina ou evidência objetiva de isquemia miocárdica não desenvolvem tal rede de vasos. Os pacientes respondem diferentemente em sua capacidade de colateralização na presença de isquemia.^3^ Uma busca começou a compreender por que...

A formação de vasos sanguíneos no sistema cardiovascular maduro ocorre por meio de três processos dinâmicos distintos: vasculogênese, angiogênese e arteriogênese. Esses sistemas são influenciados por vários fatores, incluindo sinalização e controle transcricional, mediadores solúveis e seus receptores, forças biomecânicas e hipóxia.^2^

Por exemplo, a relação entre o índice de imunoinflamação sistêmica (SII) e a circulação colateral coronariana (CCC) em pacientes com DAC estável e oclusão total crônica (OTC) foi estudada por Adali et al.^4^ Eles descobriram que um nível elevado de SII era significativamente relacionado à má circulação colateral coronariana. Portanto, um índice simples baseado em poucos exames laboratoriais pode ser muito informativo na prática clínica como preditor da capacidade de formação de colaterais de um paciente.

Agora, outro ator potencial no desenvolvimento de colateral coronariana emerge do trabalho de Akkaya et al.,^5^ publicado nesta edição dos ABC.^5^ Eles procuraram determinar a associação entre os níveis de adropina com a presença de circulação colateral em pacientes com síndrome coronariana crônica. Encontraram um aumento de 27% nos níveis médios de adropina em pacientes com boa circulação colateral coronariana em comparação com pacientes com má circulação colateral coronariana. Como os autores corretamente notaram, desenvolver uma boa rede de colaterais demanda tempo, e um único instante desse processo deve ser entendido como reflexo daquele momento específico. Se deixarmos passar tempo suficiente, talvez o quadro seja diferente.

À medida que as pesquisas avançam, novos fatores angiogênicos são relatados com maior frequência, expondo a complexidade do crescimento vascular em condições isquêmicas. Em algum momento, os cientistas sentiram-se confiantes de que poderiam recapitular o processo natural de crescimento vascular através da hiperexpressão de fatores angiogênicos específicos no novo campo da terapia gênica.^6^ Infelizmente, os ensaios clínicos randomizados com essa abordagem não tiveram o sucesso esperado, e o entusiasmo inicial foi substituído pela decepção.

O trabalho de Akkaya et al.,^5^ acrescenta à crescente literatura explorando o papel da adropina, um hormônio peptídico secretado principalmente pelo fígado, em condições clínicas tão diversas como cardiomiopatia diabética, apneia obstrutiva do sono ou doença inflamatória intestinal.^7^ Como a adropina surgiu como um componente regulatório essencial do endotélio vascular, afetando a síntese endotelial de NO, podemos prever que é uma questão de tempo para que a adropina seja considerada um alvo terapêutico na medicina cardiovascular.

Se precisarmos de outro exemplo da importância de se manter um diálogo aberto entre a ciência básica e a clínica, o artigo de Akkaya et al.,^5^ oferece exatamente isso.
