# Supplementary material for: Outro Ator no Aumento da Circulação Colateral no Coração – Outro Potencial Alvo Terapêutico na Medicina Cardiovascular?
Source: Arq Bras Cardiol. 2022 Aug 25;119(3):411–2. [Article in Portuguese] doi: 10.36660/abc.20220558 (PMC9438534; doi:10.36660/abc.20220558)
Supplement: Translation

# Translation: Another Player in Increasing Collateral Circulation in the Heart – Another Potential Therapeutic Target in Cardiovascular Medicine?

**Authors:** Luis Henrique Wolff Gowdak

**Affiliations:** 1 Brazil Instituto do Coração do Hospital das Clínicas da Faculdade de Medicina da Universidade de São Paulo, São Paulo, SP – Brazil

**Keywords:** Collateral Circulation, Neovascularization, Physiologc, Adropin, Myocardial, Ischemia, Coronary Artery Disease

Richard Lower of Amsterdam first called attention to channels connecting the right and left coronary arteries in 1669.^1^ The Swiss anatomist Albrecht von Haller demonstrated these anastomoses by dissecting the coronary arteries. So, for centuries now, the presence of a well-developed collateral network providing blood supply to an underperfused myocardium has gained significant interest in physiology and physiopathology, and later, in therapeutics. Many questions kept scientists working restlessly to unravel the key players favoring the development of collateral vessels in the heart.^2^

The coronary collateral circulation is a preformed network of immature anastomoses which connect the territory supplied by one epicardial coronary artery with that supplied by another. In 1959, Pitt et al.^1^ studied the prevalence of interarterial coronary anastomoses in seventy-five hearts obtained from an autopsy. Of the 15 normal hearts, only one (6%) was found to have such anastomoses. In those cases with occlusive coronary artery disease, myocardium fibrosis, or infarction, anastomoses were found in 75% to 100% of the cases. It was clear, from the beginning, that the presence of sustained periods of tissue ischemia was a pre-requisite to incite de establishment of collateral circulation.

On the other hand, many patients with angina or objective evidence of myocardial ischemia failed to develop such a network of vessels. Patients responded differently in their capacity for collateralization in the presence of ischemia.^3^ A quest began to comprehend why...

The formation of blood vessels in the mature cardiovascular system occurs through three distinct dynamic processes: vasculogenesis, angiogenesis, and arteriogenesis. These systems are influenced by various factors, including signaling and transcriptional control, soluble mediators and their receptors, biomechanical forces, and hypoxia.^2^

For instance, the relationship between the systemic immune-inflammation index (SII) and coronary collateral circulation (CCC) in patients with stable CAD and chronic total occlusion (CTO) was studied by Adali et al.^4^ They found that a high SII level was significantly related to poor coronary collateral circulation. Therefore, a simple index based on a few laboratory tests could be very informative in clinical practice as a predictor of a patient´s capability of collateral formation.

Now, another potential player in coronary collateral development emerges from the work of Akkaya et al.^5^ published in this issue of the ABC.^5^ They sought to determine the association between adropin levels with the presence of collateral circulation in patients with chronic coronary syndrome. They found a 27% increase in the mean adropin levels in patients with good compared to patients with poor coronary collateral circulation. As the authors correctly noted, developing a good network of collaterals demands time, and a single snapshot of this process must be understood as reflecting that particular moment. If we allow enough time to elapse, perhaps the picture will be different.

As research progresses, novel angiogenic factors are reported with increased frequency, exposing the complexity of the vascular growth in ischemic conditions. At some point, scientists felt confident they could recapitulate the natural process of vascular growth through hyperexpression of specific angiogenic factors in the new field of gene therapy.^6^ Unfortunately, randomized clinical trials with this approach did not succeed as expected, and the early enthusiasm was replaced by disappointment.

The work of Akkaya et al.^5^ adds to the growing literature exploring the role of adropin, a peptide hormone secreted primarily by the liver, in clinical conditions as diverse as diabetic cardiomyopathy, obstructive sleep apnea, or inflammatory bowel disease.^7^ Because adropin emerged as an essential regulatory component of the vascular endothelium by affecting endothelial NO synthesis, we may foresee that it is a matter of time when adropin will be considered a therapeutic target in cardiovascular medicine.

If one needs another example of the importance of keeping an open dialog between basic and clinical science, the paper by Akkaya et al.^5^ offers just that.
